# Vermont

**Published:** 1896-09

**Authors:** 


*Vermont.* The State Board of Health, working in conjunction with the Cattle Commissioners, are doing a great deal of educational and advisory work in controlling tuberculosis among their live-stock.

